# Bisbenzamidine derivative, pentamidine represses DNA damage response through inhibition of histone H2A acetylation

**DOI:** 10.1186/1476-4598-9-34

**Published:** 2010-02-09

**Authors:** Junya Kobayashi, Akihiro Kato, Yosuke Ota, Reiko Ohba, Kenshi Komatsu

**Affiliations:** 1 Department of Genome Repair Dynamics, Radiation Biology Center, Kyoto University, Kyoto 606-8501, Japan; 2 National Institute of Genetics, Yata 1111, Mishima, Sizuoka 411-8540, Japan

## Abstract

**Background:**

MRE11 is an important nuclease which functions in the end-resection step of homologous recombination (HR) repair of DNA double-strand breaks (DSBs). As MRE11-deficient ATLD cells exhibit hyper radio-sensitivity and impaired DSB repair, MRE11 inhibitors could possibly function as potent radio-sensitizers. Therefore, we investigated whether a bisbenzamidine derivative, pentamidine, which can inhibit endoexonuclease activity, might influence DSB-induced damage responses *via *inhibition of MRE11.

**Results:**

We first clarified that pentamidine inhibited MRE11 nuclease activity and also reduced ATM kinase activity in vitro. Pentamidine increased the radio-sensitivity of HeLa cells, suggesting that this compound could possibly influence DNA damage response factors in vivo. Indeed, we found that pentamidine reduced the accumulation of γ-H2AX, NBS1 and phospho-ATM at the sites of DSBs. Furthermore, pentamidine decreased HR activity *in vivo*. Pentamidine was found to inhibit the acetylation of histone H2A which could contribute both to inhibition of IR-induced focus formation and HR repair. These results suggest that pentamidine might exert its effects by inhibiting histone acetyltransferases. We found that pentamidine repressed the activity of Tip60 acetyltransferase which is known to acetylate histone H2A and that knockdown of Tip60 by siRNA reduced HR activity.

**Conclusion:**

These results indicate that inhibition of Tip60 as well as hMRE11 nuclease by pentamidine underlies the radiosensitizing effects of this compound making it an excellent sensitizer for radiotherapy or chemotherapy.

## Background

DNA double-strand breaks (DSBs) are generated by exposure to ionizing radiation, DNA damaging agents such as bleomycin or neocarzinostatin, or due to the stalling or collapse of DNA replication forks. As unrepaired DSBs induce genome instability and promote apoptosis or tumorigenesis, cells recognize DSBs immediately and activate cell cycle checkpoints and DNA repair mechanisms. Hence, the generation of DSBs by exposure to ionizing radiation (IR) could induce cell death in tumor cells and the inhibition of DSB repair activity in tumors might lead to efficient radiotherapy. The generation of DSBs triggers the re-localization of many DNA damage response (DDR) proteins such as MRE11/NBS1/RAD50, MDC1, 53BP1 and BRCA1 to nuclear foci that co-localize with γ-H2AX [1-5]. H2AX is rapidly phosphorylated at DSB sites and phosphorylated H2AX (γ-H2AX) interacts with NBS1, MDC1 and BRCA1, thereby promoting their accumulation at DSBs [1,6]. Hence, H2AX-knockout cells are deficient in the formation of DSB-induced nuclear foci of several DDR proteins such as NBS1 [2,6,7]. Furthermore, H2AX-knockout cells are defective in homologous recombination (HR) repair [8]. Both H2AX+/- and H2AX-/- mouse thymocytes show an increase in chromosomal aberrations [9,10]. These facts indicate that γ-H2AX-depedent foci formation could be important for DSB repair, particularly HR repair, and genome stability.

MRE11 nuclease is a key factor in DSB damage response and functions as both a single- and double-stranded DNA endonuclease as well as 3'->5' exonuclease [11,12]. It has been reported that this nuclease activity is indispensable for homologous recombination, both during DSB repair and during meiotic recombination using yeast cell lines lacking functional Mre11 [13,14]. Mutations in the hMRE11 gene result in Ataxia Telangiectasia-like disorder (AT-LD) syndrome. Both AT-LD patient cells and ATM-defective Ataxia Telagiectasia patients cells show similar phenotypes such as radio-resistant DNA synthesis, radiation hyper-sensitivity and genome instability [15-17]. hMRE11 forms a complex with NBS1 and hRAD50 and this complex displays DNA binding and tethering activities as well as nuclease activity. This complex has been shown to function in DNA double-strand break repair by HR in mammals [18,19]. Moreover, efficient HR repair requires IR-induced focus formation (recruitment) of the NBS1/hMRE11/hRAD50 complex at DNA damage sites [20]. Hence, the genomic instability in AT-LD patients could be due to the defect in HR. Therefore, the inhibition of hMRE11 nuclease activity or recruitment of this complex may result in radiosensitization.

The bisbenzamidine derivative, pentamidine, has been one of the most successful agents against eukaryotic parasites and has been used clinically against trypanosomiasis, leishmananiasis, and Pneumocystis carinii for over 70 years [21-23]. Pentamidine enters parasite cells rapidly and appear first in the kinetoplast that contains the mitochondrial DNA of the parasite. With time it is also generally seen in the cell nucleus but significant amounts are not observed in the cytoplasm. Pentamidin is capable of binding to the minor groove of double-strand DNA but not single-strand DNA and inhibits protein synthesis, DNA synthesis and the activity of endo-exonuclease in Pneumocystis carinii [24]. Further, DNA and protein synthesis in human tumor also decreased by pentamidine treatment [25]. Recently, it was reported that pentamidine also inhibited human endo-exonuclease activity in vitro and induced cell death in several tumor cells efficiently [26]. Although it is unclear as to whether pentamidine might inhibit other nucleases such as hMRE11, the effect of pentamidine on hMRE11 could potentially lead to anti-tumorigenic effects or effective radiotherapy.

In this paper, we first demonstrate the inhibitory effect of pentamidine to hMRE11 nuclease activity in vitro. We also show that pentamidine increases the radio-sensitivity of HeLa cells and represses IR-induced focus formation of γ-H2AX and NBS1. Furthermore, pentamidine reduces the HR activity and acetylation of histone H2A, mediated by Tip60 histone acetytransferase (HAT). Moreover, pentamidine reduces the HAT activity of Tip60 in vitro. We discuss that such these novel inhibitory effects of pentamidine on hMRE11 and Tip60 opens up important therapeutic and radiosensitzing options for cancer therapy.

## Results

### Pentamidine inhibits in vitro MRE11 nuclease activity and ATM kinase activity

**Figure 1:**
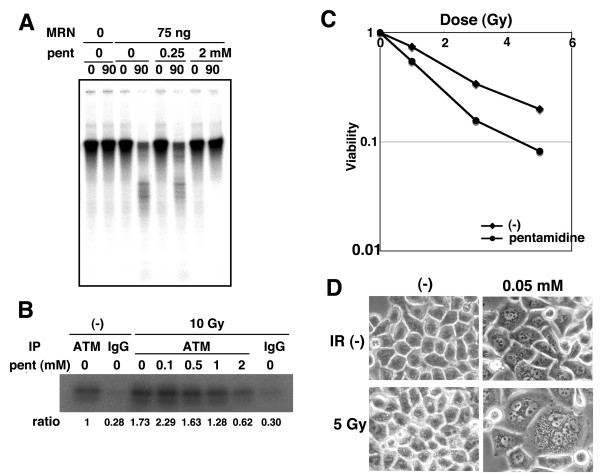
**The inhibitory effect of pentamidine on an hMRE11 nuclease and an ATM kinase**. (A) Pentamidine inhibited hMRE11 nuclease. hMRE11 nuclease assay was performed using recombinant MRN complex. Indicated amount of pentamidine is added to the reaction mixture. (B) Pentamidine inhibited ATM kinase. Normal lymphoblastoid cells (GM2184) were irradiated by 10 Gy of γ-irradiation and the kinase activity assay was performed using immuno-complex by anti-ATM antibody or control rabbit IgG from these cells. Indicated concentration of pentamidine is added to the reaction mixture. (C) Pentamidine treatment increased radiation sensitivity. HeLa cells were irradiated with indicated dose of γ-ray with or without pentamidine (0.05 mM) and the viability of their cells were analyzed by colony forming assays. (D) Pentamidine treatment induced multinuclear formation. HeLa cells were irradiated with 5 Gy of γ-ray with or without pentamidine (0.05 mM) and cell morphology was observed after 24 hours.

It has been previously reported that some dicationic diaryfurans such as pentamidine inhibit the endo-exonuclease in Pneumocystus carinii [24]. As human endo-exonuclease activity was also reported to be repressed pentamidine in vitro [26], we investigated whether pentamidine was capable of inhibiting hMRE11 nuclease activity in vitro (Fig. 1A). Digestion of the hMRE11 substrate was completed by 90 mins. Importantly, upon addition of pentamidine (2 mM) substrate digestion was almost completely inhibited even after 90 mins. Thus, pentamidine could inhibit hMRE11 nuclease activity in vitro. The MRN complex is known to bind with ATM kinase directly and this interaction is essential for the activation of ATM [27]. Hence, we next examined whether pentamidine could influence ATM kinase activity in vitro (Fig. 1B). Immunoprecipitated ATM from un-irradiated cells exhibited low levels of p53 phosphorylation activity and this was increased 1.73 times upon irradiation. However, the addition of pentamidine (1 or 2 mM) resulted in an obvious decrease in ATM kinase activity. Thus, pentamidine reduced both hMRE11 nuclease and ATM kinase activities in vitro. As these activities are very important for IR-induced DNA damage responses and subsequent fate of the cells, pentamidine would be expected to increase radio-sensitivity. Therefore, the effect of pentamidine treatment on radio-sensitivity was examined in HeLa cells (Fig. 1C). The viability after 5 Gy of irradiation is about 0.2, but the addition of pentamidine (0.05 mM) decreased the cell viability to approximately 0.08. Further, the treatment of 0.1 mM pentamidine also increased radio-sensitivity in HeLa cells (Additional file 1, Fig. S1). Moreover, 0.05 mM of pentamidine treatment induced a few multinucleated cells without irradiation, and irradiation increased the number of multinucleated cells (Fig. 1D). Multinucleation upon pentamidine treatment might be a consequence of the cells proceeding into mitosis without appropriate DSB repair or cell cycle checkpoint implementation resulting in mitotic catastrophe.

### Pentamidine represses the formation of DNA-damage responsive foci upon irradiation

**Figure 2:**
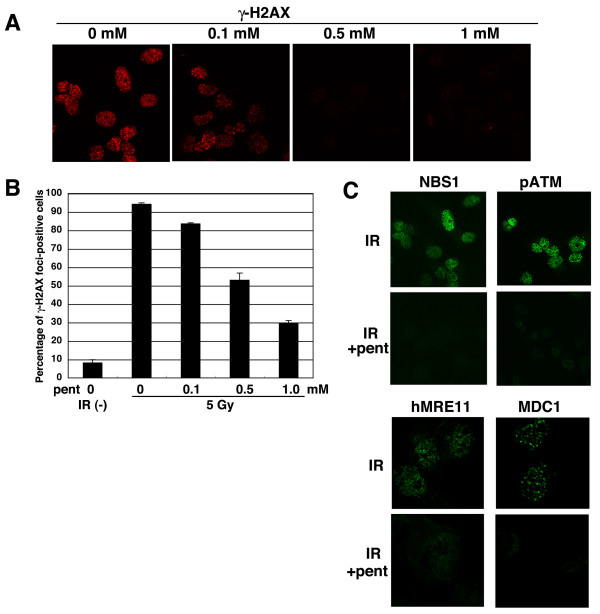
**The effect of pentamidine on IR-induced cellular response**. (A) Pentamidine repressed γ-H2AX foci formation. MRC5SV cells were irradiated with 5 Gy of γ-ray with or without pre-treatment of pentamidine (indicated concentrations, 30 minutes). After 30 minutes, their cells were fixed and immuno-staining was performed using anti-γ-H2AX. Percentage of γ-H2AX foci-positive cell was shown in (B). (C) Pentamidine repressed focus formation of DNA damage-related factors. MRC5SV cells were irradiated with 5 Gy of γ-ray with or without pre-treatment of pentamidine (0.5 mM, 30 minutes). After 30 minutes, their cells were fixed and immuno-staining was performed using anti-NBS1, anti-phospho-ATM, anti-hMRE11 and anti-MDC1 antibodies.

**Figure 3:**
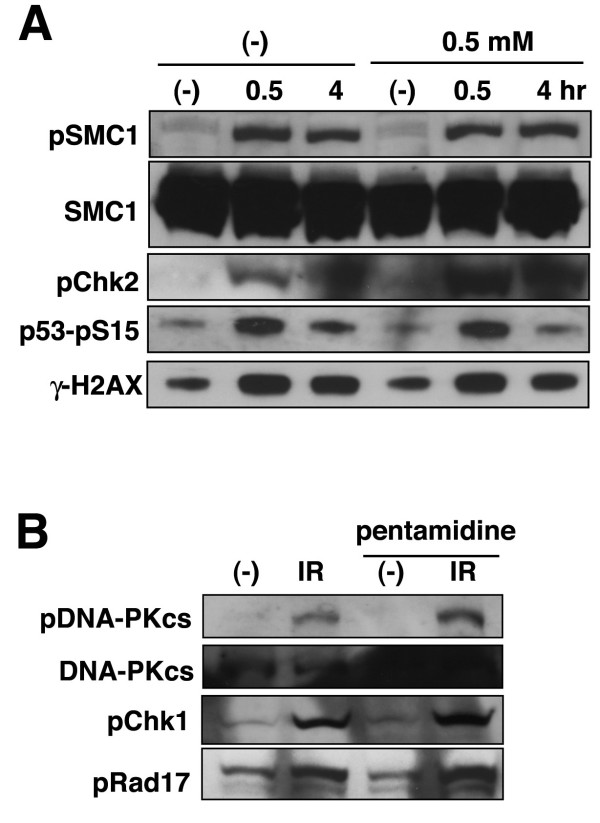
**The effect of pentamidine on ATM/ATR/DNA-PK-dependent phosphorylation**. (A) Pentamidine did not disturb ATM-dependent phosphorylation. MRC5SV cells were irradiated by 5 Gy of γ-ray with or without pre-treatment of pentamidine (0.5 mM, 30 minutes). These cells were harvested at 0.5 hour after IR and analyzed by Western blot using indicated antibodies. (B) Pentamidine did not disturb ATR or DNA-PK-dependent phosphorylation. MRC5SV were irradiated by 5 Gy of γ-ray with or without pre-treatment of pentamidine (0.5 mM, 1 hour). These cells were harvested at 0.5 hour after IR and analyzed by Western blot using indicated antibodies.

Fig. 1 indicated that very low concentration (0.05 mM) of pentamidine influenced the radio-sensitivity and multi-nuclear formation in vivo, although more than 0.5 mM of pentamidine is required for inhibition to hMRE11 activity or ATM kinase activity in vitro. As hMRE11/ATM are some of the earliest responders to DSBs and trigger many aspects of DDR, pentamidine may influence the function of other factors in DNA damage response. The accumulation of several DDR proteins into "foci" following IR is early and important event in DNA damage-induced cellular response, particularly γ-H2AX foci, which are formed before most DNA damage responses. Therefore, we investigated the effect of pentamidine on γ-H2AX focus formation following IR (Fig. 2AB). Without pentamidine treatment, most cells exhibited γ-H2AX focus formation after an exposure to 5 Gy of γ-ray. Pre-treatment with pentamidine (more than 0.5 mM) resulted in a striking reduction in focus formation. Previously, we reported that the IR-induced focus formation of NBS1, which forms a complex with hMRE11 and hRAD50, is dependent on γ-H2AX [1]; therefore, we verified the inhibitory effect of pentamidine on the formation of NBS1 foci following irradiation (Fig. 2C and Additional file 1, Fig. S2 and S3). Similarly to γ-H2AX foci, IR-induced NBS1 focus formation was decreased by pre-treatment of pentamidine. Pentamidine also repressed focus formation by hMRE11, MDC1 and phospho-ATM, whose accumulation at DSBs is dependent on γ-H2AX or NBS1 (Fig. 2C). Recently, Chromatin-immunoprecipitation (ChIP) has been effectively used to clarify the recruitment (accumulation) of DNA damage-related factors to DSB sites [28]. Usually, the recruitment of factors that accumulated as IR-induced foci, is also detectable by ChIP assay. When we examined the recruitment of γ-H2AX or NBS1 near DSB sites by ChIP assay, these recruitments were detected without pentamidine treatment (Additional file 1, Fig. S4). However, pre-treatment with pentamidine repressed these recruitments (γ-H2AX: 0 and 4 kb; NBS1: 0 kb), which is consistent with the results of focus formation in Fig. 2ABC. However, the accumulation of γ-H2AX at 1 kb distance was not repressed by pentamidine. As the formation of γ-H2AX is composed by two steps; ATM/NBS1-dependent primary phosphorylation and subsequent MDC1-dependent accumulation [7]. Hence, inhibitory effect of pentamidine might be different between two steps. As Fig. 1B showed that pentamidine could inhibit ATM kinase activity and Fig. 2AB showed that pentamidine reduced ATM-dependent γ-H2AX focus formation, we investigated whether pentamidine could repress ATM-dependent DDR phosphorylation events (Fig. 3A). Irradiation with γ-rays (5 Gy) induced phosphorylation of SMC1, Chk2, and p53, for which ATM is the responsible kinase. However, 0.5 mM of pentamidine treatment did not reduce these phosphorylation. Moreover, the treatment of 1 mM of pentamidine, which can diminish almost γ-H2AX focus formation, did not influence these phosphorylation in both MRC5SV and HeLa cells (Additional file 1, Fig. S5AB). However, 2 mM of pentamidine treatment reduced auto-phosphorylation of ATM and SMC1 phosphorylation (Additional file 1, Fig. S5C), which is consistent with the inhibitory effect on ATM kinase activity in vitro (Fig. 1B). Moreover, low concentration of pentamidine also showed no effect on DNA-PK or ATR-dependent phosphorylations (Fig. 3B). Taken together, low concentration of pentamidine might repress IR-induced foci formation and accumulation of proteins such as γ-H2AX independently of inhibition to ATM kinase in vivo.

### Pentamidine represses both homologous recombination repair and histone acetylation

**Figure 4:**
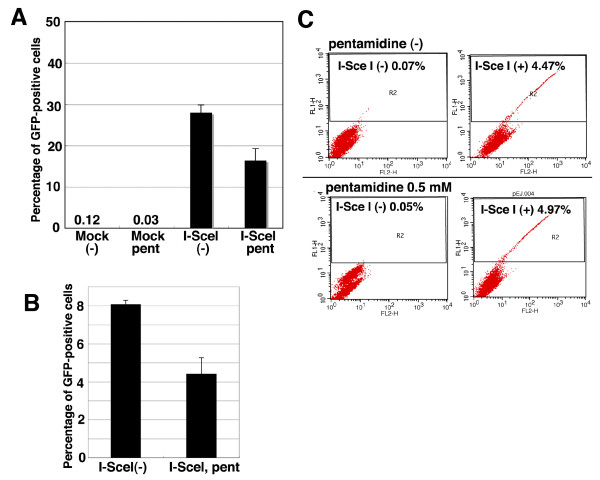
**The effect of pentamidine on DNA double-strand break repair (A)(B) Pentamidine decreased homologous recombination activity in DR-GFP assay**. I-SceI expression plasmids were introduced to M5D (A) or HeLa-DRGFP cells (B) by an electroporation with or without pre-treatment of pentamidine (0.5 mM, 1 hour). After 3 days GFP-positive cells, induced via HR pathway, were analyzed by flowcytometer as described in Materials and Methods. (C) Pentamidine did not disturb NHEJ activity. I-SceI expression plasmids were introduced to MRC5-pEJ cells by electroporation with or without pre-treatment of pentamidine (0.5 mM, 1 hour). After 3 days GFP-positive cells, induced through NHEJ pathway, were analyzed by flowcytometer as described in Materials and Methods.

**Figure 5:**
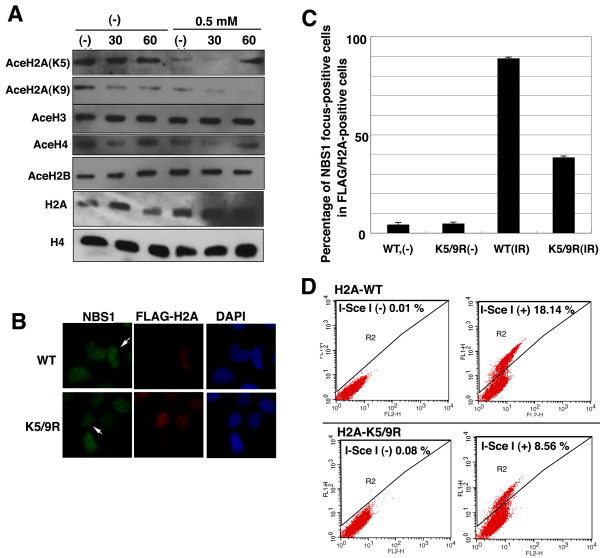
**Pentamidine might affect DNA damage response through acetylation of histone H2A**. (A) The effect of pentamidine on histone acetylation. MRC5SV cells were irradiated with 5 Gy of γ-ray with or without pre-treatment of pentamidine (0.5 mM, 30 minutes). These cells were harvested at indicated times after IR and analyzed by Western blot using indicated antibodies. (B) Acetylation of histone H2A is required for IR-induced foci formation. H2A-WT or H2A-K5/9R-expressing cells were generated as described in Materials and Methods. These cells were irradiated with 5 Gy of γ-ray. After 30 minutes, their cells were fixed and immuno-staining was performed using anti-NBS1 and anti-FLAG antibodies. Percentage of NBS1 foci-positive cells was shown in (C). (D) HR activity in H2A (K5/9R)-expressing cells. I-SceI expression plasmids were introduced into H2A-WT or H2A-K5/9R- expressing cells by electroporation. After 2 days GFP-positive cells, induced through HR pathway, were analyzed by flowcytometer.

Various DNA damage-related proteins form nuclear foci at DSB sites, and many of them, such as the MRN complex, γ-H2AX, BRCA1, Rad51, are known to function in homologous recombination repair [8,20,29]. As Fig. 2 indicated that pentamidine reduced their focus formation, we next examined whether pentamidine repressed HR repair. When we used the DR-GFP system developed by the Jasin laboratory [30] to estimate HR activity in MRC5SV cells, the generation of DSB by an expression of I-SceI restrection enzyme induced approximate 28% of GFP-positive cells (Fig. 4A). Pre-treatment with pentamidine reduced GFP-positive cells to 16% after I-SceI introduction. In the case of HeLa cells, similar effect of pentamidine was observed (Fig. 4B). We also estimated the effect of pentamidine on NHEJ repair, but the frequency of GFP-positive cells via NHEJ pathway was unchanged with or without pentamidine treatment (Fig. 4C). These results suggest that prentamidine could repress HR repair activity. Recently, the importance of histone modification, particularly, its acetylation in HR repair has been reported [31]. Using the DR-GFP assay we found that the histone acetytransferase (HAT)-specific inhibitors anacardic acid and curcumin reduced HR activity, but Trichostatin A (histone deacetylase inhibitor) did not influence the HR activity (Additional file 1, Fig. S6), suggesting that histone acetylation is indispensable for HR. Surprisingly, pentamidine reduced the acetylation of histone H2A at Lys5 dramatically, and the acetylation of H2A at Lys9 was also decreased, although γ-irradiation did not increase the acetylation of these histones (Fig. 5A). As this acetylation was expected to have an important role in DNA damage responses, we generated exogenous FLAG-H2AX (WT or K5/9R mutants)-expressing MRC5SV cells. K5/9R-H2A-expressing cells exhibited a decrease in NBS1-focus formation following IR (Fig. 5BC). Moreover, the HR activity in K5/9R-H2A-expressing cells was much less than that in WT-H2A-expressing cells (Fig. 5D). Taken together, the acetylation of H2A at Lys5 and Lys9 appears to play an important role in HR repair and pentamidine might influence the IR-induced focus formation and HR repair through a repression of histone H2A acetylation.

### Pentamidine inhibits Histone acetyltransferase, Tip60

Fig. 5 indicates the possibility that pentamidine might inhibit the acetylation of histone H2A in addition to inhibiting the activities of hMRE11 and ATM (Fig. 1AB). Hence, the decrease in histone H2A acetylation might be an indirect effect of pentamidine through ATM or hMRE11 activity. However, ATM inhibitors did not influence the acetylation of H2A with or without irradiation (Additional file 1, Fig. S7A), although it reduced SMC1 phosphorylation. hMRE11-deficient ATLD cells showed normal levels of acetylation (Additional file 1, Fig. S7B), suggesting that hMRE11 nuclease activity is dispensable for an acetylation of histone H2A.

**Figure 6:**
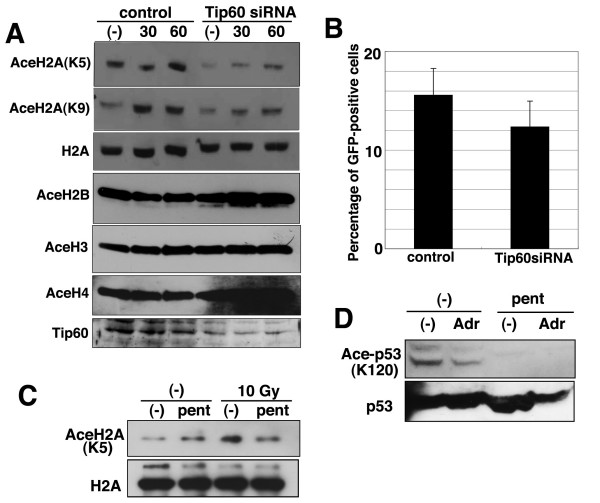
**Tip60-knockdown also repressed both HR activity and acetylation of histone H2A**. (A) Tip60-knockdown reduced acetylation of histone H2A. MRC5SV cells were transfected by Tip60 siRNA. After 2 days, these cells were irradiated by 5 Gy of γ-ray and were harvested at indicated times after IR and analyzed by Western blot using indicated antibodies. (B) HR activity in Tip60-knockdowned cells. M5D cells were transfected by Tip60 siRNA. After 2 days, I-SceI expression plasmids were introduced by electroporation. After 3 days, GFP-positive cells were analyzed by flowcytometer. (C) The effect of pentamidine on HAT activity of Tip60. Whole cell extract was prepared from irradiated or un-irradiated HeLa cells. And then, Tip60-dependent HAT activity was measured with or without pentamidine (5 μM) as described in Material and Methods. (D) Pentamidine repressed Tip60-dependent acetylation of p53. MRC5SV cells were added by 0.5 mM adriamycin with or without pre-treatment of pentamidine (0.5 mM, 30 minutes). These cells were harvested after 4 hours and analyzed by Western blot using anti-acetylated p53 antibody.

Tip60 histone acetyltransferase is reported to be responsible for histone acetylation and can acetylate histone H2A in vitro [32]. Further, several lines of evidence suggests that Tip60 might function in DSB damage response [31,33]. Therefore, we examined whether the repression of Tip60 by siRNA result in an effect that is similar to pentamidine treatment. Tip60-knock-down cells showed clear reduction of H2A acetylation at Lys5 and Lys9, but did not influence the acetylation of H3, H2B, and H4 (Fig. 6A). Moreover, the repression of Tip60 also reduced HR activity in DR-GFP system (Fig. 6B). These results suggest that Tip60 is a crucial acetyltransferase for histone H2A and plays a role in HR repair through the acetylation oh histone H2A. We next investigated if pentamidine directly inhibit Tip60 activity in vitro (Fig. 6C). Immunoprecipitated Tip60 could acetylate recombinant H2A in vitro and 10 Gy of irradiation increased this activity. However, the addition of pentamidine abolished the induction of Tip60 activity. Tip60 is reported to acetylate p53 at Lys120 as well as hisotone H2A at Lys5 and Lys9 [34]. Pentamidine treatment also reduced Tip60-dependent acetylation of p53 in MRC5SV cells (Fig. 6D), suggesting that pentamidine could inhibit Tip60 acetyltransferase acitivity in vivo. Taken together, pentamidine could repress histone acetylation through direct inhibition of Tip60 histone acetyltransferase, and this repression might leads to a decrease in HR repair activity and cell viability after irradiation.

## Discussion

We identified here that pentamidine is a novel inhibitor of hMRE11 nuclease (Fig. 1A). Pentamidine also repressed ATM kinase activity in vitro (Fig. 1B). Low concentrations of pentamidine enhanced radio-sensitivity of HeLa cells (Fig. 1C), suggesting that pentamidine might influence other factors in DNA damage response. Pentamidine reduced IR-induced focus formation of DDR proteins such as γ-H2AX and NBS1, although a remarkable effect on ATM-dependent phosphorylations was not observed (Fig. 2 and 3). Pentamidine also repressed HR activity but not NHEJ (Fig 4). Furthemore, pentamidine reduced acetylation of histone H2A (Fig. 5A), which is suggested to influence IR-induced focus formation and HR repair. H2A mutated at acetylation sites decreased both IR-induced focus formation of NBS1 and HR activity (Fig. 5BCD). Knockdown of Tip60, which is known as a histone acetyl transferase, resulted in effects on H2A acetylation and HR activity similarly to that seen upon pentamidine treatment (Fig. 6AB). Moreover, pentamidine reduced HAT activity of Tip60 in vitro and Tip60-dependent acetylation of p53 in vivo (Fig. 6CD), suggesting that the inhibitory effects of pentamidine on DDR may be mediated, at least in part, by inhibitin of Tip60. However, the effects of Tip60-knockdown were less pronounced than those by pentamidine treatment (Fig. 6B). Taken together, pentamidine might influence IR-induced DNA damage response through the inhibitory effect on not only Tip60, but also hMRE11 and ATM.

Pentamidine is known to be as one of the most effective agents against Pneumocystis carinii. Pentamidin is capable of binding to the minor groove of double-strand DNA and inhibits protein synthesis, DNA synthesis and the activity of endo-exonuclease in Pneumocystis carinii [24]. Pentamidine also repressed Saccharomyces cerevisiae RNC1/TRM2 endo-exonuclease, which displayed 5'->3' exonuclease activity on double-strand DNA and endonuclease activity on single-strand DNA in vitro [35]. Recently, it was reported that pentamidine also inhibited human endo-exonuclease activity in vitro [26]. These observations suggest that pentamidine might show an inhibitory effect on other nuclease such as hMRE11 in mammalian cells. Indeed, our results demonstrate that addition of 2 mM pentamidine abolished hMRE11 nucelase activity in vitro (Fig. 1A), suggesting that pentamidine could be used as an effective inhibitor in human cells. We also showed that pentamidine reduced ATM kinase acitivity. As ATM requires MRN complex for optimal activation [27], the effect of pentamidine on ATM kinase activity might occur via repression of hMRE11 nuclease by pentamidine. Future experiments will be aimed at clarifying whether pentamidine reduces ATM kinase activity directly.

Recently, several reports suggest a tight relationship between histone acetylation and DNA damage response. Tamburini and Tyler showed that acetylation of histone H3 and H4 increased at HO endonuclease-restricted DSB sites in yeasts by ChIP assay and also showed that the histone acetyltransferases Gcn5 and Esa1 were recruited at these DSB sites [36]. Downs and his colleagues also reported that Arp4, a subunit of NuA4 HAT complex interacts with phosphorylated H2A directly and Arp4 was recruited to HO-related DSB sites [37]. Further, human homologous of NuA4 HAT, Tip60 interact with hitstone H2AX and could acetylated it, and also showed that acetylation of histone H2AX increased in response to DSB damage [33]. Furthermore, the strains of S. pombe or S. cerevisieae expressing mutated H3 or H4 at acetylation sites or HAT-deficient yeasts increased the sensitivity to DNA damaging agents in yeast and dominant-negative Tip60-expressing HeLa cells showed the delay of DSB repair following IR by comet assay [32,36]. These reports suggest that acetylation of histon is important for DNA damage response and, perhaps, DNA repair. This is consistent with our results showing that the H2A-K5/9R (acetylation site mutant)-expressing cells showed a decrease in HR repair activity (Fig. 5D) and Tip60-knockdown cells also reduced the HR activity (Fig. 6B). We also indicated that pentamidine treatment and the H2A (K5/9R)-expressing cells repressed both focus formation and HR (Figs 2ABC, 4AB and 5BCD), suggesting that focus formation of DNA damage-related factors such as NBS1 and γ-H2AX contributes to HR repair pathway through histone acetylation. In fact, a partner protein of Tip60, TRAPP-knockout mouse cells showed a decrease in accumulation of histone acetylation at DSB sites, IR-induced focus formation such as BRCA1 or Rad51 and HR activity [31]. Moreover, we reported that the defect of MRE11 focus formation in mutated NBS1-expressing cells leads to reduction of HR activity [20]. Thus, focus formation of DNA damage response factors could be closely related with homologous recombination repair. Moreover, pentamidine might influence HR repair through repression of histone acetylation, related with IR-induced focus formation.

So far, it was reported that the bisbenzamidine derivative, pentamidine showed the growth repression against several tumor cells such as MCF7 and HeLa cells via repression of endo-exonuclease activity [26]. The paper also showed that normal human diploid fibroblats are not sensitive to pentamidine and pentamidine displayed an inhibitory effect on tumor growth in mouse model. These facts suggest that pentamidine might be an effective anti-tumor reagent. Moreover, pentamidine has a synergistic inhibitory effect to tumor growth with mitomycin C or other anti-growth reagent, suggesting that pentamidine may show a synergistic action with ionizing radiation [38,39]. In fact, Fig. 1C shows the expected effect with irradiation of γ-ray, and hence lower concentration (0.05 mM) of pentamidine might be an effective sensitizer for radiotherapy. However, much higher concentration of pentamidine was required for inhibitory effects on Mre11 nuclease and ATM kinase activity in vitro (Fig. 1AB), while middle range concentration of pentamidine reduced Tip60 activity in vitro and in vivo (Fig. 6CD). These results suggest that the effect of pentamidine (low concentration) as a radiation sensitizer could be due to an inhibitory effect on Tip60. In order to use pentamidine or other deamidine analogues with radiotherapy or chemotherapy, we need to clarify all the targets of these reagents in DNA damage response and the underlying mechanisms in detail.

## Conclusion

In conclusion we have found the inhibitory effect of pentamidine on Tip60 acetyltransferase and ATM kinase. This effect caused a repression of DDR factors' focus formation and an enhancement of radio-sensitivity particularly via a reduction of histone H2A acetylation. We also found that both the acetylation of histone H2A and the role Tip60 are important for IR-induced focus formation and homologous recombination repair. Therefore, the further research of pentamidine could find an excellent sensitizer for radiotherapy or chemotherapy.

## Materials and methods

### Pentamidine

Pentamidine (pentamidine isethionate; Chugai Co., Japan) is dissolved in sterile water and stored at 4°C.

### Cell lines

MRC5SV cells were SV40-transformed MRC5 human fibroblasts [7,40]. NBS fibroblast cell line, GM07166VA7, was established by SV40-transformation of GM07166 cells, which were provided from NIGMS Cell Repository [40]. AT-LD fibroblast cell line, ATLD2SV was established by SV40-transformation of ATLD2 primary fibroblasts, which were supplied by Dr Y. Shiloh [41]. Normal lymphoblastoid cell line, GM2184 was also obtained from NIGMS Cell Repository. H2A (WT) or H2A (K5/9R)-expressing M5D cells were generated by a transfection of pCMV-Tag2 plasmid (Stratagene) inserted with human H2A cDNA. After G418 selection, their expression in isolated cells were confirmed by Western blot analysis.

### Antibodies

Phospho-ATM (S1981) rabbit polyclonal antibody (Epitomics Inc.), phospho-SMC1 (S966) rabbit polyclonal and SMC1 rabbit polyclonal, MDC1 rabbit polyclonal antibodies (Bethyl Laboratories Inc.), phospho-p53 (S15) mouse monoclonal, phospho-Chk2 (T68) rabbit polyclonal antibodies (Cell Signaling Technology), hMre11 rabbit polyclonal and Nbs1 rabbit polyclonal antibodies (Novus Biologicals), and Chk2 rabbit polyclonal and anti Tip60 rabbit polyclonal antibodies (Santa Cruz Biotechnology), and γ-H2AX mouse monoclonal acetyl H2A (K5) rabbit polyclonal, acetyl H2A (K9) rabbit polyclonal, acetyl H2B rabbit polyclonal, acetyl H3 rabbit polyclonal, acetyl H4 rabbit polyclonal, histone H2A rabbit polyclonal and histone H4 rabbit polyclonal antibodies (Millipore Co.), and p53-acetyl-K120 mouse monoclonal (BioAcademia) were used for Western blot analysis or immuno-staining.

### SiRNA knock-down experiments

Sub-confluent cells, seeded to culture dishes the day before, were transfected by Tip 60 (Be-Bridge International Inc.) or negative (Be-Bridge International Inc.) siRNA using lipofectamine 2000 (Invitrogen Life Technology). After 2 days, these cells were re-seeded in proper culture dishes. Next day, these cells were used for Western blot or DR-GFP analysis.

### Western Blot analysis

Western blot analysis was carried out as described previously [1]. Target proteins were detected with primary antibodies, mentioned above, and HRP-conjugated anti-rabbit IgG or anti-mouse IgG antibodies (GE Helthcare), and then visualized with an ECL plus chemiluminescence system (GE Helthcare).

### Immunofluorescent staining

Immunofluorescent staining was carried out as described previously [1]. Alexa-488-conjugated anti-rabbit IgG (Molecular Probes) or Alexa-594-conjugated anti-mouse IgG (Molecular Probes) were used for visualization of foci of target protein.

### ATM kinase assay

ATM kinase assay was carried out as described previously [7]. ATM was immunoprecipitated with an anti-ATM antibody (Calbiochem). Phosphorylation of p53 (substrate) by immunoprecipitated ATM in vitro in the presence of [γ-^32^P]-ATP was detected by BAS2000 (FUJI Film Co.).

### MRE11 nuclease assay

MRE11 nuclease assay was performed as previously reported [11,12]. MRN complex proteins for this assay are purified as previously reported [12]. MRE11 nuclease reactions (containing 25 mM MOPS [pH 7.0], 50 mM NaCl, 2 mM DTT, 1 mM MnCl_2_, 0.1 pmole of DNA substrate labeled by [γ-^32^P]-ATP and 150 ng of MRN complexes), were performed in a volume of 10 μl, and were incubated at 37°C for 30 min. Reactions were stopped by the addition of EDTA and SDS to final concentrations of 10 mM and 0.2%, respectively. Each reaction was dried down, resuspended in 5 μl of formamide loading buffer, and then loaded onto a sequencing gel containing 15%-22.5% acrylamide and 7 M. After the run, each gel was analyzed with BASS2000.

### Cell survival assay

For radiation sensitivity assays, the cells were trypsinized and irradiated with 1, 3, or 5 Gy of ^60^Co γ-ray at a dose rate of 1.1 Gy/min. Immediately after irradiation, cells were plated into 100-mm dishes at such a cell density that 30-40 cells would survive, and incubated for 14 days. The dishes were fixed with ethanol, stained with 4% Giemsa, and the number of colonies were counted. Surviving fractions were calculated by comparing the number of colonies formed by irradiated cells with the number of colonies formed by non-irradiated control cells. Each result represents an average value from 3 experiments.

### Homologous Recombination assay (DR-GFP) assay

DR-GFP was performed as previously reported [20,30]. To measure the repair of an I-SceI-generated DSBs, 50 μg of the I-SceI expression vector (pCBASce) was introduced to 1000000 M5D cells, by electroporation (GenePulser; BIO-RAD). To determine the amount of HR repair, the percentage of cells, that were GFP-positive, was quantified by flow cytometry 3 days after electroporation with FACScalibur (Becton Dickinson).

### NHEJ assay

NHEJ was performed as previously reported [42]. To measure the repair of an I-SceI-generated DSBs via NHEJ pathway, 50 mg of the I-SceI expression vector pCBASce was introduced to 1000000 MRC5SV-pEJ cells, by electroporation (BIO-RAD). To determine the amount of NHEJ repair, the percentage of GFP-positive cells was quantitated with flow cytometric analysis at 3 days after electroporation by FACScalibur (Becton Dickinson).

### In vitro histone acetylation assay by Tip60

DNA damage-treated or untreated cells were lysed in IP buffer (150 mM sodium chloride, 10 mM Tris/HCl [pH7.4] and 0.5% NP40) containing a protease inhibitor cocktail (Roch) and sodium orthovanadate for 15 min. Lysates were centrifuged at 20,000 × g for 30 min to remove un-solubulized debris. Lysates were pre-cleared with protein A-Sepharose beads (GE healthcare), and immunoprecipitation was performed by incubating the samples with anti-Tip60 antibody (Santa Cruz) at 4°C for 1 hour. After washing by IP buffer, these immunoprecipitants were used for in vitro histone acetylation assay. Reaction of Tip60-dependent histone acetylation (50 mM Tris/HCl [pH 8.0], 10% grycerol, 0.1 mM EDTA, 1 mM DTT, 1 mM PMSF, 10 mM sodium Butyrate, 20 mM Acetyl CoA, 10 μg of histone H2A) was performed at 37°C for 30 minutes and the reaction was stopped by addition of SDS-PAGE loading buffer. The actylated histone H2A in the reaction was detected by Western blot analysis using anti-acetyl H2A (K9) rabbit polyclonal antibody.

## Competing interests

The authors declare that they have no competing intereats.

## Authors' contributions

JK designed the experiments, carried out a large part of experiments and drafted the manuscript. AK performed the MRE11 nuclease assay. YO performed the experiments about immunofluorescent staining and western blot in part. RO carried out ChIP analysis. KK supervised the project and commented on the manuscript. All authors read and approved the final manuscript.
